# Electrical Heating Performance of Graphene/PLA-Based Various Types of Auxetic Patterns and Its Composite Cotton Fabric Manufactured by CFDM 3D Printer

**DOI:** 10.3390/polym13122010

**Published:** 2021-06-19

**Authors:** Hyelim Kim, Sunhee Lee

**Affiliations:** 1 Research Institute of Convergence Design, Dong-A University, Busan 49315, Korea; hyelim1221@gmail.com; 2 Department of Fashion Design, Dong-A University, Busan 49315, Korea

**Keywords:** conveyor-fused deposition modeling 3D printer, graphene/PLA, auxetic pattern, unit cell, electrical heating performance

## Abstract

To evaluate the electrical heating performance by auxetic pattern, re-entrant honeycomb (RE), chiral truss (CT), honeycomb (HN), and truss (TR), using graphene/PLA (Polylactic acid) filament, were manufactured by CFDM (conveyor fused deposition modelling) 3D printer. In addition, HN and TR, which was indicated to have an excellent electrical heating property, were selected to verify the feasibility of applying fabric heating elements. The result of morphology was that the number of struts constituting the unit cell and the connected points were TR < HN < CT < RE. It was also influenced by the surface resistivity and electrical heating performance. RE, which has the highest number of struts constituting the unit cell and the relative density, had the highest value of surface resistivity, and the lowest value was found in the opposite TR. In the electrical heating performance of samples, the heat distribution of RE was limited even when the applied voltage was increased. However, HN and TR were diffused throughout the sample. In addition, the surface temperature of RE, CT, HN, and TR was about 72.4 °C, 83.1 °C, 94.9 °C, and 85.9, respectively as applied at 30 V. When the HN and TR were printed on cotton fabric, the surface resistivity of HN/cotton and TR/cotton was about 10^3^ Ω/sq, which showed conductive material. The results of electrical heating properties indicated that the heat distribution of HN/cotton showed only in the region where power was supplied, but the TR/cotton was gradually expanded and presented stable electric heating properties. When 30 V was applied, the surface temperature of both samples showed more than 80 °C, and the shape was maintained stably due to the high thermal conductivity of the cotton fabric. Therefore, this study ensured that HN and TR show excellent electrical heating performance among four types of auxetic patterns with continuity.

## 1. Introduction

A cellular structure is defined as an object’s architecture as formed by and array of spatial arrangement of unit cells with edges and faces. These structures are networks of interconnected struts or walls with porosities and cellular solids available in both two and three dimensions [1,2]. Recently, studies on structures such as re-entrant honeycomb and chiral truss, which are auxetic structures showing negative Poisson’s ratio properties, have also been conducted [3,4,5]. Auxetic structure material is defined as the material having a negative Poisson’s ratio which has a characteristic of expanding or contracting the transverse side under uniaxial stretching or compression, unlike a general material having a positive Poisson’s ratio [3]. Auxetic structures and materials exhibit a superior mechanical property of negative Poisson’s ratio such as static modulus, energy absorption, shear resistance, indentation resistance, synclastic curvature, crash worthiness, and sound absorption, etc. It is known that these auxetic structure are classified by geometric forms such as re-entrant, chiral, and rotating polygon structures [4,5]. It is known that these macro-scale mechanical properties of auxetic structures originate from their different small-scale topology and mainly used applications that are also different depending on their geometrical features of structure [5].

3D printing technology that can print prototypes by designing complex and various types of structures through 3D modeling has been used in various fields [6]. Using 3D printing technology, the production of lattices has created many opportunities for the creation of new functional lattices such as those with an auxetic structure [7,8,9]. Most of the previous studies on 3D printed cellular or auxetic structure have used FDM technology for fabricating cellular or auxetic structure using PLA (Polylactic acid), ABS (acrylonitrile butadiene styrene), and TPU (Thermoplastic polyurethane) materials and mainly reported on improving mechanical properties such as compressive and energy absorption properties according to relative density or topologies of the unit cell design [10,11,12]. Filaments in which carbon nanomaterials are added in a thermoplastic polymer are being developed in order to improve the mechanical properties, commercialization, and development of filaments; this is also important since electrical properties can also be exhibited [13,14,15,16,17]. It has been applied to conductive circuits, electrodes, or sensors for 3D printed flexible electronic devices [18,19]. Several studies have been reported to develop a conductive circuits or sensor by 3D printing a cellular and auxetic structure with a conductive material [20,21,22,23,24]. Ronca et al. [20] have fabricated 3D printed TPU/graphene cellular structures for strain sensitivity. It was reported that it was possible to manufacture flexible sensors due to the porous structure, especially the schwarz structure was shown to have an excellent property because of the higher size of trabeculae connecting the porosity. Wang et al. [23] have reported the development of a 3D printed stretchable temperature sensor consisting of cellular graphene/polydimethylsiloxane (PDMS) composite. In a study of temperature-sensing properties of three cellular structure, the result was found that all samples present more stable sensitivities than the solid sample, and the grid structure sample delivered more stable sensing performance. 

Previous studies on the 3D printed cellular structure by conductive materials [20,21,22,23,24] have mainly dealt with the manufacture and development of strain, compressive, and temperature sensors due to the structural characteristics having porosity. However, the research on the analysis of electrical heating properties and development of electrical heating elements using 3D printed auxetic patterns or structures printed with conductive filaments is still insufficient. Accordingly, our research team continuously studied the electrical heating element by conductive filament by CFDM 3D printer [25,26]. In our previous study, the honeycomb structure, which is a continuous structure using two types of carbon nano-based conductive filaments, was printed by CFDM 3D printer, and the electrical and electrical heating properties of the sample were excellent at the CD direction [27]. In addition, a CFDM 3D printed horseshoe pattern with graphene/PLA was investigated by 3D printing direction. It was confirmed that the sample with 3D printed at 0°, which is same as the 3D printing direction, was improved in regard to its electrical heating properties due to the horizontal direction having less contact resistance than the diagonal and vertical directions [28,29]. Thus, the purpose of this present study is to provide a basic study for applying the electrical heating element by 3D printer and conductive filament to safety and protective clothing and devices. Accordingly, the aim is to analyze the electrical heating performance of CFDM 3D printing samples by the auxetic pattern having a continuous structure. First, four different auxetic patterns—re-entrant honeycomb, chiral truss, honeycomb, and truss patterns—were designed and 3D modeled and then printed by a CFDM 3D printer. After that, the surface morphology, electrical property, and electrical heating property depending on the auxetic patterns were confirmed. Finally, the honeycomb and truss structures, which showed excellent electrical heating properties, were printed on cotton fabric to confirm their applicability as fabric heating elements. 

## 2. Materials and Methods

### 2.1. Materials

The conductive graphene (GR)/PLA filament (Blackmagic 3D Ltd., New York, NY, USA) used in this study is the same material as used in our previous study [29]. It is for FDM 3D printing with 1.75 mm of diameter and 0.6 Ω/cm of conductivity [14]. The filament was stored in the desiccator at a standard condition before being used for analysis and 3D printing. 

### 2.2. Preparation of Graphene/PLA-Based CFDM 3D-Printed Auxetic Patterns

#### 2.2.1. Design and 3D Modelling of Auxetic Pattern

In this study, to confirm the electric heating performance according to the structural form of the auxetic pattern, 4 types known as auxetic patterns were selected [3,4,5]. Table 1 shows sample codes and 3D modeling images for each of the four auxetic patterns. For designing 4 types of auxetic patterns of re-entrant honeycomb (RE), chiral truss (CT), honeycomb (HN) and truss (TR), a unit cell for repeating was designed with the horizontal and vertical dimensions of 10 mm × 10 mm and line of thickness of 2 mm. The four types of auxetic pattern of 50 mm × 50 mm were finally designed by arranging 5 repeating unit cells of each designed pattern horizontally and vertically. After that, 3D modeling with a size of 50 mm × 50 mm × 1 mm was created using the 123D design program. Finally, the obtained 3D modeling file was converted into g-code file in Blackbelt Cura 3.6.0 program for preparing 3D printing.

#### 2.2.2. Graphene/PLA-Based CFDM 3D Printing Conditions

For printing, a conveyor type FDM (CFDM) 3D printer (Blackbelt 3D B.V., Belfeld, the Netherlands) was used, as it was in our previous studies [26,29]. 3D printing conditions such as temperature of nozzle and bed, printing speed and infill were controlled. To print the four-types of CFDM 3D printed auxetic patterns using GR/PLA filament, the CFDM 3D printing conditions were controlled as follows: 220 °C of nozzle temperature, 50 °C of bed temperature, 30 mm/s of printing speed, 22.5 mm/s of infill speed, and 100% of infill. 

#### 2.2.3. Preparation of Graphene/PLA–based CFDM 3D Printed Auxetic Pattern on Cotton Fabric

To confirm the possibility of preparing the fabric heating element using Graphene/PLA-based CFDM 3D printed auxetic pattern among 4 types of structure, TR and HN patterns were 3D printed on cotton fabric. In this study, the substrate fabric selected was a cotton fabric with plain structure, having a thickness of 0.27 ± 0.01 mm, a weight per unit area of 0.019 ± 0.001 g/cm^2^, and a density of warp and weft yarns of 72/inch and 64/inch, respectively. It was prepared at a size of 60 mm × 60 mm. Then, the substrate fabric was put on the conveyor type belt. Finally, TR and HN were printed on the substrate fabric with a thickness of 1 mm using a GR/PLA filament and CFDM 3D printer. Each sample named as TR/cotton and HN/cotton, respectively.

### 2.3. Characterization

#### 2.3.1. Morphology

To analyze of Graphene/PLA-based CFDM 3D printed auxetic patterns and its composite fabrics, the morphology was measured by digital camera (HDR-CX550, Sony, corp., Tokyo, Japan), and fabric image analysis microscopy (NT 100, Nextec, Gunpo, Korea) at ×6.5 magnification.

#### 2.3.2. Surface Resistivity

To investigate the electrical property, the surface resistivity of Graphene/PLA-based CFDM 3D printed samples by different auxetic patterns was measured with multimeter (ST850A, Saehan Tester, Co Ltd., Busan, Korea) based on the AATCC-76 method. The electrical resistance (R) was measured at least five times at edges of four-types of sample. The surface resistivity (R_s_) was calculated as Equation (1):

R_s_(Ω/sq) = (W/D) × R, (1)

where R is the resistance (Ω) measured by the multimeter, W is the width (mm) of the sample, and D (mm) is the distance between the two electrodes.

#### 2.3.3. Electrical Heating Performance

For confirming the electrical heating property of Graphene/PLA-based CFDM 3D printed samples and its composite cotton fabrics by different auxetic patterns, the surface temperature was measured by different voltages while using a DC power supply (CPS-2450B, CHUNGPA EMT Co. Ltd., Bucheon, Korea) and thermal imaging camera (FLIR i5, FLIR Systems INC., Wilsonville, OR, USA). At first, copper tape was attached both edges of each sample. After then, the alligator clips were placed to both edges of samples and voltage was applied from 5 to 30 V with 5 V (DC) intervals for 3 min [29]. The heat distribution, surface temperature and current of each sample were measured by the thermal imaging camera after applying different voltages.

## 3. Results and Discussion

### 3.1. CFDM 3D Printed GR/PLA Samples with Various Auxetic Patterns

#### 3.1.1. Morphology

In this study, to compare the macroscopic forms of different types of auxetic patterns, each pattern was analyzed using a digital image and fabric image analysis system. Table 2 shows the morphology of CFDM 3D printed samples with various auxetic patterns. As shown in the first line of Table 2, which gives digital images of low magnification, it was confirmed that three-dimensional RE, CT, HN, and TR patterns using GR/PLA could be printed using a CFDM 3D printer. The dimensions of 4 types of CFDM 3D printed samples were 50 mm × 50 mm × 1 mm. As according to the auxetic patterns, although the unit cells were modeled identically, the relative density of CFDM 3D printed samples was found to have the highest RE. In high magnification digital images, it was shown that each unit cell of auxetic pattern and the GR/PLA filaments uniformly stacked for each layer according to the nozzle direction. In addition, the CFDM 3D printed samples with 4 types of different auxetic pattern were 3D modeled in the same printing direction, and accordingly, it was confirmed through the surface morphology that the GR/PLA filaments were stacked in the same 3D printed direction. Therefore, it was confirmed that four types of CFDM 3D printed samples can be printed according to the auxetic pattern with GR/PLA filaments. 

#### 3.1.2. Surface Resistivity

**Figure 1:**
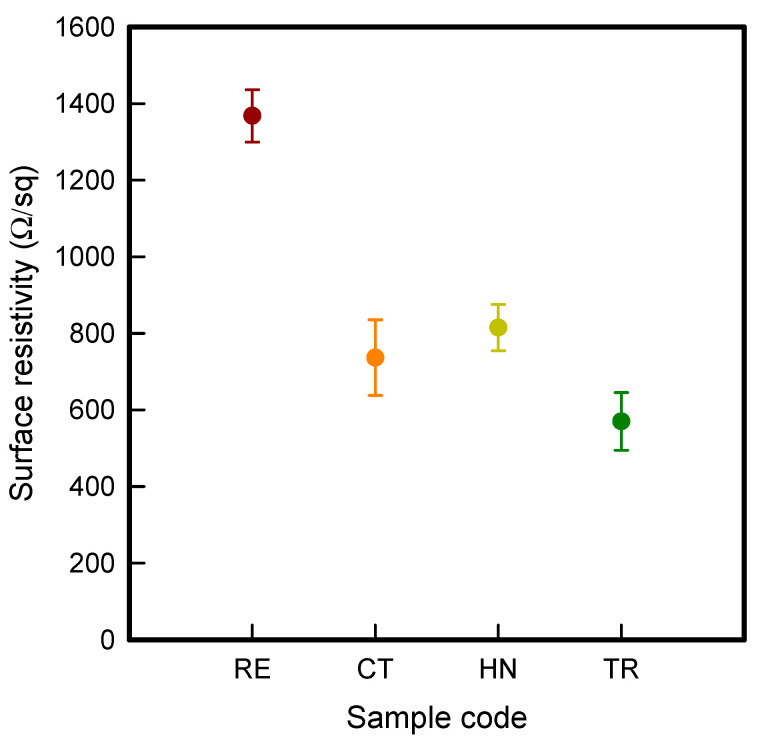
Surface resistivity of 4 types of CFDM 3D printed graphene/PLA auxetic patterns.

To investigate the electrical property by the influence of auxetic patterns with different unit cell such as RE, CT, HN, and TR, Figure 1 presents the surface resistivity of CFDM 3D printed samples with various auxetic patterns. The surface resistivity values of RE, CT, HN, and TR samples were 1.4 × 10^3^ ± 6.9 × 10^1^ Ω/sq, 7.4 × 10^2^ ± 7.3 × 10^1^ Ω/sq, 9.2 × 10^2^ ± 6.0 × 10^1^ Ω/sq, and 5.7 × 10^2^ ± 7.5 × 10^1^ Ω/sq, respectively. It was shown that the surface resistivity was increased in the order of TR < CT < HN < RE. In our previous study [29], CFDM 3D printed horseshoe pattern with GR/PLA filament was shown over than 10^3^ Ω/sq. In this present study, the results of surface resistivity of 4 types of CFDM 3D printed samples with various auxetic pattern were declined 10 times more than the 3D printed horseshoe pattern. It was because the auxetic pattern has a continuous structure that is connected to each strut, and it was confirmed that graphene, which is a filler in the polymer matrix, could also be made as a continuous conductive path. 

In addition, it was confirmed that the surface resistivity values depending on the auxetic pattern. Generally, it is known that the resistance increases as the length increases due to the resistance (*R*) being proportional to the length (*L*) and inversely proportional to the cross-section area (*A*) in the formula of *R* = *L/A* [24]. Filipowska et al. [24] have reported that when the line width of electro-conductive paths and patterns increased, the electro-conductivity increased, whereas the line length decreased, and the electro-conductivity also decreased. In addition, the previous study that produced fabric heating elements coated by graphene/polymer was designed and manufactured using stripe and horseshoe patterns [25]. As a result, the electrical resistivity was more increased than the stripe pattern type sample due to the generation of resistivity at the curved region of the horseshoe pattern type sample. Based on these results, the number and length of each strut constituting the unit cells of four auxetic patterns was shown as TR < CT < HN < RE sample. As shown in Table 2, it is shown that this is due to the highest relative density of 3D printed sample indicated in RE sample. Therefore, it was confirmed that the surface resistivity of the TR with the lower strut length of the unit cell was the lowest while forming a uniform symmetrical structure.

#### 3.1.3. Electrical Heating Performance

To confirm the electrical heating property of CFDM 3D printed samples by different auxetic patterns, Table 3 represents the IR images of 4 types of CFDM 3D printed of auxetic patterns with various applied voltages for 3 min to temperature stabilize. The surface temperature of CFDM 3D printed samples with RE, CT, HN, and TR showed the tendency of the increase with increasing applied voltages. In this study, to confirm the applicability of the fabric heating element, the shape stability and performance maintenance at the temperature that rises according to the applied voltage had to be confirmed. When the surface temperature of the CFDM 3D printed samples was over 60 °C or more, the samples were indicated to have a characteristic of being flexible by heat; in the same way as in our previous study, this is due to the glass temperature of PLA, which has been indicated to be around 59 °C [26]. Therefore, it was confirmed to be shape stable when the surface temperature was at 60 °C or less. In addition, 4 types of CFDM 3D printed samples with different auxetic patterns were shown to have an expanded heating area according to the increase in the applied voltage, from 5 V to 30 V. At each auxetic pattern, it was confirmed that the electrical heating distribution appeared uniformly in the order of RE < CT < HN < TR. This can be explained by the Joule heating phenomenon [27]. Joule heating is a phenomenon that occurs when an electrical current is passed through a material with an electrical resistance. The resistance that is inherent to the material leads to a conversion of electrical energy to thermal energy [27,28]. Therefore, the resistive heating is caused by the collision of moving electrons with atoms that are constituents of the main material, and accordingly, it was confirmed that the heating area showed gradually increase. In addition, as previously confirmed in the morphology and surface resistivity, in the case of the RE pattern having a relatively high density among 4 types of 3D printed samples and the resistance being high, it was shown that the electrical heating properties at the location where power is supplied were not expanded and exhibited overall. On the other hand, it was found that CT, HN, and TR represented a more uniform heat distribution than RE. According to a previous study [28], the electrical resistivity was increased at curved region of horseshoe pattern type sample, and accordingly, it was shown that the difference of surface temperature between straight and curved region. Thus, the heat distribution was indicated in order of RE < CT < HN < TR.

**Figure 2:**
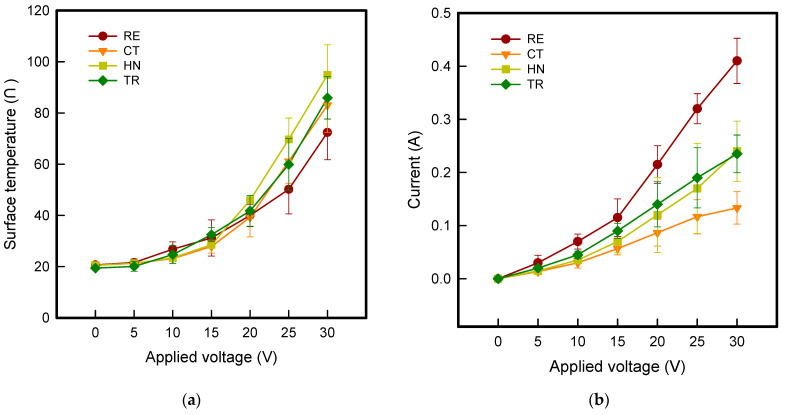
(**a**) Surface temperature and (**b**) *I-V* curve of 4 types of CFDM 3D printed graphene/PLA auxetic patterns.

Based on the result of thermal images, Figure 2 indicates the surface temperature and current of CFDM 3D printed samples with various types of auxetic patterns. As shown in Figure 2a, when the applied voltage increased, CFDM 3D printed samples with various types of auxetics patterns also increased linearly. As applied voltage at 30 V, the surface temperature of RE, CT, HN, and TR samples were indicated 72.4 ± 10.6 °C, 83.1 ± 10.2 °C, 94.9 ± 11.5 °C, and 85.9 ± 8.3 °C, respectively. In addition, the current-voltage (*I-V*) curve of CFDM 3D printed samples with various auxetic patterns is shown in Figure 2b. The current of RE, CT, HN, and TR samples tended to gradually increase. When the voltage was applied from 5 V to 30 V, the current value of RE, CT, HN, and TR samples were shown as from 0.00 to 0.41 A, from 0.00 A to 0.13 A, 0.00 A to 0.24 A, and from 0.00 A to 0.24 A, respectively. Additionally, CFDM 3D printed samples were influenced by types of auxetic patterns and the current were increased in order of CT < HN = TR < RE at maximum voltage of 30 V. In case of RE sample, it shows more than two times than other samples. This is confirmed by the high relative density of the RE sample, as confirmed in the morphological characteristics identified above, which consumes a lot of current compared to other samples. 

However, as seen in the IR image, it was confirmed that the high density caused disorder in the power supply, and thus the whole surface was not expanded the heat distribution because the electrode was located, and thus the average surface temperature was low. On the other hand, CT sample showed the lowest current value but more heat distribution than RE sample. It was confirmed that the amount of graphene present in the sample was small and easy to flow the resistive heating because the relative density was low. In the case of HN and TR samples, it was confirmed that the current increased linearly and showed similar current values compared to other samples. It was confirmed because structurally, struts are uniformly symmetrically connected in all directions, so surface resistance is low and current flows smoothly when power is supplied. Through the above results, the current value was highest in the RE sample, but the HN and TR samples exhibited the highest surface temperature with uniform heat distribution in the HN and TR samples where the current value increased linearly and uniformly. Therefore, it was confirmed that the HN and TR samples were shown the best electrical heating properties.

### 3.2. CFDM 3D Printed GR/PLA Auxetic Pattern/Cotton Composite Fabric

#### 3.2.1. Surface Resistivity

Table 4 presents the surface resistivity values of CFDM 3D printed GR/PLA-based HN and TR on cotton fabric. In this section, the patterns of HN and TR which has the excellent electrical properties and electrical heating properties were CFDM 3D printed on cotton fabric to confirm the comparison between patterns and pattern/cotton composite fabrics by GR/PLA. The surface resistivity value of HN/cotton and TR/cotton were 9.1 × 10^2^ ± 9.8 Ω/sq and 7.7 × 10^2^ ± 9.7 Ω/sq, respectively. When comparing the surface resistivity values of patterns of HN and TR, the HN/cotton and TR/cotton values were increased about 2.0 × 10^2^ Ω/sq. It was confirmed that the increase of surface resistivity of HN/cotton and TR/cotton due to use the cotton fabric as a substrate material which is a general insulation material, whereas HN and TR made of only GR/PLA. In addition, TR/cotton indicated less surface resistivity value than HN/cotton same as pattern tendency. It is because the length of the struts constituting the unit cell of the TR is shorter than that of the HN at the same relative density, as shown in Table 2. However, in general, since the conductive material is known to exhibit a resistance of 10^1^–10^6^ Ω/sq, thus, both HN/cotton and TR/cotton have been confirmed to have electrical properties and conductive material. 

#### 3.2.2. Electrical Heating Performance

To investigate the possibility for applying the fabric heating element using CFDM 3D printed auxetic patterns, the electrical heating properties of HN/cotton and TR/cotton were measured. The surface temperature was measured by thermal image camera as applied voltages from 5 V to 30 V for 3 min. 

Table 5 represents the IR images of CFDM 3D printed GR/PLA-based HN and TR patterns on cotton fabric. In the case of HN/cotton and TR/cotton, it was confirmed that the shapes were maintained stably, unlike the CFDM 3D printed GR/PLA-based pattern sample, which showed a phenomenon of being flexible at 60 °C or higher. It was confirmed that because of the high thermal conductivity, cotton fabric was used as the substrate material. As seen in Table 5, HN/cotton and TR/cotton showed the heat distribution expands as the applied voltage increases. In case of HN/cotton, the heat distribution was generated in only the middle of the sample where the power supply was. On the other hand, the heat distribution of TR/cotton was shown as more enlarged and with a more uniform area than HN/cotton. It was confirmed that the TR/cotton showed a lower surface resistivity value than the HN/cotton, as previously mentioned in the surface resistivity results. Structurally, the truss unit cell is more symmetrical than the honeycomb unit cell, and the number of connected struts is small, so it is confirmed that the conductive path in the TR can be made more easily. Therefore, this study has confirmed that the heating distribution was limited because the resistivity increased in the connected region as the number of struts increased. Accordingly, it was found that the heat distribution of CFDM 3D printed GR/PLA-based TR/cotton was improved with the TR with the smallest strut number in the unit cell. 

**Figure 3:**
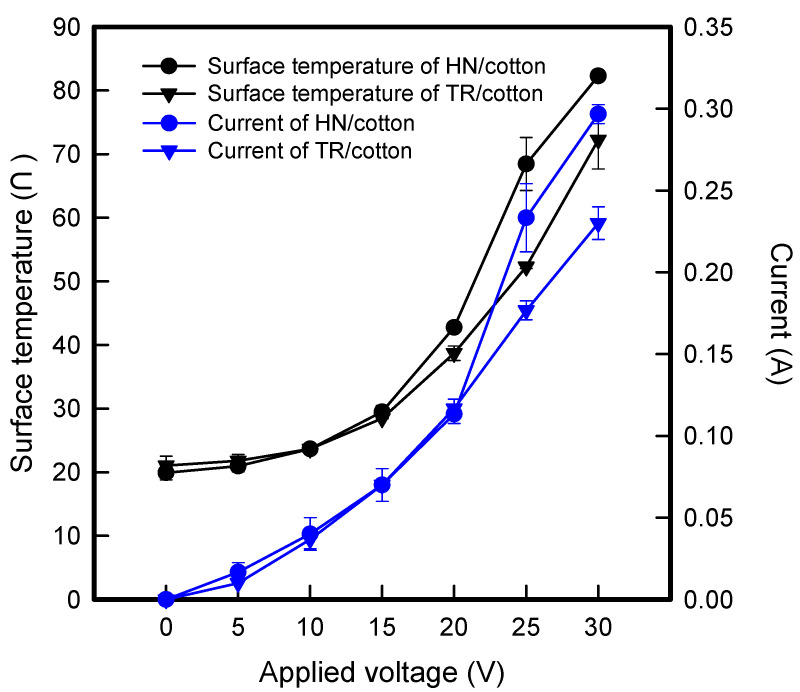
Surface temperature and Current-voltage (I-V) curve CFDM 3D printed graphene/PLA auxetic patterns/cotton composite fabric with honeycomb and truss pattern.

Figure 3 shows the surface temperatures and current-voltage curve of HN/cotton and TR/cotton by different applying voltages. The surface temperature and current of HN/cotton increased linearly to 20 V, and then increased rapidly from 20 V or more. When the applied voltage was at 30 V, the mean temperature of HN/cotton was 80.6 ± 5.8 °C, whereas the maximum temperature of HN/cotton was indicated over 150 °C, which is a similar value of T_m_ of PLA. On the other hand, TR/cotton surface temperature and current tended to increase linearly as the applied voltage increased from 0 V to 30 V. When the applied voltage at 30 V, the mean and maximum temperatures of TR/cotton were about 71.9 °C and 130.0 °C, respectively. In addition, the current value of HN/cotton was indicated to be higher than the TR/cotton, which was about 0.07 A when the applied voltage was at 30 V. At that time, the current values of HN/cotton and TR/cotton were about 0.30 A and 0.23 A, respectively. In general, power of heat (*Q*) is calculated by Joule’s heating according to Equation (2):

*Q = I*^2^*Rt*, (2)

where *I* is current, *R* is resistance, and *t* is period of the time. Since the current and surface resistivity of the HN/cotton sample was indicated higher values than TR/cotton sample, it was confirmed that the power value of electrical heating was large. In addition, as mentioned above, in the case of TR/cotton sample, since there are few structurally connected regions, it has a low surface resistivity. At that time, as the power is supplied, heat distribution and temperature are improved even with a lower current than the HN/cotton sample. On the other hand, it was confirmed that the HN/cotton has a higher resistivity than TR/cotton and that the heat distribution is limited as power is supplied. The previous study reported that the electrical heating property improves as the area of the fabric heating element decreases [24]. As the voltage is applied, the friction of the electric charge to be moved increases and can quickly emit heat so that it can exhibit a higher electrical heating property. In this study, since HN/cotton gave the same voltage in a limited area, it seems that the current and surface temperature were excessive at 20 V compared to TR/cotton where the heat distribution is continuously diffused. According to the above results, it was confirmed that TR/cotton showing stable electrical heating performance is suitable as a fabric heating element.

## 4. Conclusions

In this study, four types with continuity of CFDM 3D printed auxetic patterns using graphene/PLA filament were manufactured to evaluate the electrical property and electrical heating property by unit cell shape. In addition, HN and TR patterns, which indicate having excellent electrical and electrical heating properties, were selected to verify the feasibility of applying fabric heating elements. 

The result of morphology found that the number of struts constituting the unit cell and the connected points were increased as following: TR < HN < CT < RE. It was confirmed that these results also influenced the surface resistivity and the electrical heating properties. In the case of the surface resistivity, RE with the highest number of struts constituting the unit cell and the relative density of the pattern was highest, and the lowest value was found in the opposite TR. In the result of electrical heating property, the heat distribution of RE was limited even when the applied voltage was increased, but the heat distribution of HN and TR patterns was diffused throughout the sample. When the applied voltage was at 30 V, the surface temperature of RE, CT, HN, and TR samples was about 72.4 °C, 83.1 °C, 94.9 °C, and 85.9 °C, respectively. As the results of the HN and TR pattern printed on cotton fabric, the surface resistivity of HN/cotton and TR/cotton were higher than those of the pattern sample printed only with the GR/PLA composite material, but the value of 10^3^ Ω/sq showed that it was a conductive material. The results of electrical heating properties indicated that HN/cotton showed a heat distribution only in the region where power was supplied, and the temperature and current increased rapidly at 20 V or more. However, in TR/cotton, the surface temperature and current were linearly increased as the applied voltage increased, and the heat distribution was gradually expanded to show stable electric heating properties. When 30 V was applied, both samples showed a surface temperature of more than 80 °C, and the shape was maintained stably due to the high thermal conductivity properties of the cotton fabric. 

Therefore, in this study, four types of auxetic patterns with continuity were printed on a cotton fabric as a GR/PLA-based filament using a CFDM 3D printer capable of continuous processing to confirm the possibility of use and application as a fabric heating element. In the future, this is expected to enable mass production of fabric heating elements that can be applied in various industries including safety and protective area equipment using various substrate fabrics, conductive filaments and CFDM 3D printing technology, a new manufacturing process technology.

## Figures and Tables

**Table 1:** Sample code and 3D modelling of various auxetic patterns.

Type	Sample Code	Unit	Pattern	.stl	.gcode
Re-entrant honeycomb	RE	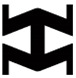	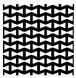	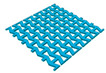	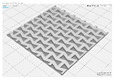
Chiral truss	CT	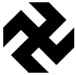	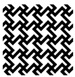	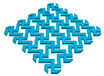	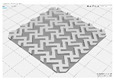
Honeycomb	HN	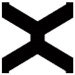	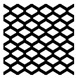	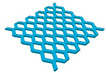	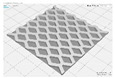
Truss	TR	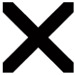	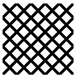	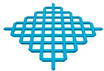	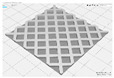

**Table 2:** Morphology of 4 types of CFDM 3D printed GR/PLA auxetic patterns.

Magnification	Sample Code			
	RE	CT	HN	TR
Digital Image	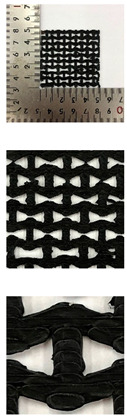	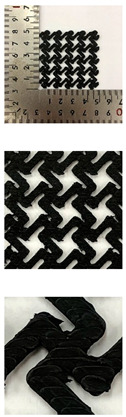	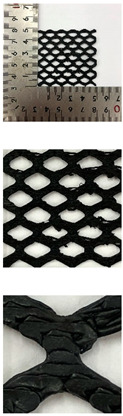	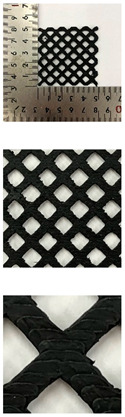
×6.5	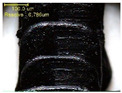	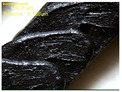	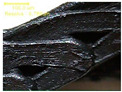	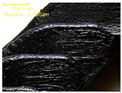

**Table 3:** IR images of 4 types of CFDM 3D printed graphene/PLA auxetic patterns with various applied voltages.

**RE**	**Applied Voltage (V)**					
	**5**	**10**	**15**	**20**	**25**	**30**
IR Image	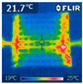	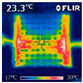	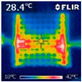	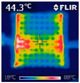	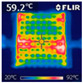	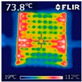
Temp. (°C)	21.6 ± 1.4	26.8 ± 3.9	31.2 ± 7.0	40.0 ± 4.4	55.2 ± 4.6	72.4 ± 10.6
Current (A)	0.03 ± 0.01	0.07 ± 0.01	0.12 ± 0.04	0.22 ± 0.04	0.32 ± 0.03	0.41 ± 0.04
**CT**	**Applied Voltage (V)**					
	**5**	**10**	**15**	**20**	**25**	**30**
IR Image	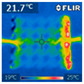	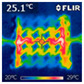	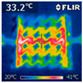	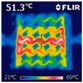	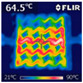	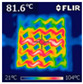
Temp. (°C)	21.3 ± 0.6	23.2 ± 1.0	31.2 ± 2.6	44.7 ± 7.6	61.5 ± 8.6	83.1 ± 10.2
Current (A)	0.01 ± 0.01	0.03 ± 0.01	0.06 ± 0.02	0.09 ± 0.03	0.12 ± 0.03	0.13 ± 0.03
**HN**	**Applied Voltage (V)**					
	**5**	**10**	**15**	**20**	**25**	**30**
IR Image	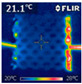	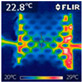	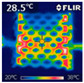	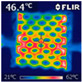	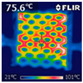	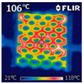
Temp. (°C)	21.7 ± 0.3	25.1 ± 0.5	29.6 ± 1.4	50.0 ± 6.1	69.7 ± 8.4	94.9 ± 11.5
Current (A)	0.02 ± 0.01	0.04 ± 0.01	0.08 ± 0.02	0.13 ± 0.05	0.17 ± 0.06	0.24 ± 0.06
**TR**	**Applied Voltage (V)**					
	**5**	**10**	**15**	**20**	**25**	**30**
IR Image	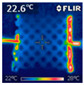	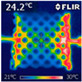	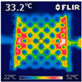	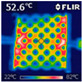	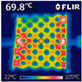	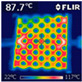
Temp. (°C)	21.1 ± 1.9	24.7 ± 3.5	32.5 ± 2.7	41.8 ± 5.9	59.9 ± 10.1	85.9 ± 8.3
Current (A)	0.02 ± 0.00	0.05 ± 0.01	0.09 ± 0.01	0.14 ± 0.04	0.19 ± 0.06	0.24 ± 0.04

**Table 4:** Surface resistivity of CFDM 3D GR/PLA-based HN and TR on cotton fabric.

Sample Code	Surface Resistivity (Ω/sq)
HN/cotton	912.2 ± 98.0
TR/cotton	769.1 ± 96.8

**Table 5:** IR images of CFDM 3D printed graphene/PLA auxetic pattern/cotton composite fabric by honeycomb and truss pattern.

**HN/Cotton**	**Applied Voltage (V)**					
	**5**	**10**	**15**	**20**	**25**	**30**
IR Image	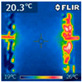	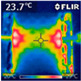	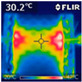	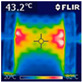	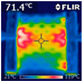	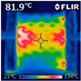
Temp. (°C)	21.5 ± 0.4	25.3 ± 1.3	32.3 ± 1.4	45.2 ± 2.9	62.5 ± 5.3	80.6 ± 5.8
Current (A)	0.02 ± 0.01	0.04 ± 0.01	0.07 ± 0.01	0.11 ± 0.01	0.23 ± 0.02	0.30 ± 0.00
**TR/Cotton**	**Applied Voltage (V)**					
	**5**	**10**	**15**	**20**	**25**	**30**
IR Image	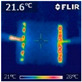	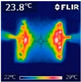	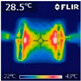	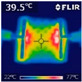	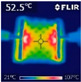	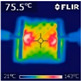
Temp. (°C)	21.1 ± 1.9	24.7 ± 3.5	32.5 ± 2.7	41.8 ± 5.9	59.9 ± 10.1	71.9 ± 8.3
Current (A)	0.01 ± 0.00	0.04 ± 0.01	0.07 ± 0.00	0.12 ± 0.01	0.18 ± 0.01	0.23 ± 0.01

## References

[1] Wu Y., Yang L. (2020). The effect of unit cell size and topology on tensile failure behavior of 2D lattice structures. Int. J. Mech. Sci..

[2] Nagesha K., Dhinakaran V., Shree M.V., Kumar K.P.M., Chalawadi D., Sathish T. (2020). Review on characterization and impacts of the lattice structure in additive manufacturing. Mater. Today Proc..

[3] Ren X., Das R., Tran P., Ngo T.C., Xie Y.M. (2018). Auxetic metamaterials and structures: A review. Smart Mater. Struct..

[4] Saxena K.K., Das R., Calius R.E.P. (2016). Three decades of auxetics research—Materials with negative Poisson’s ratio: A review. Adv. Eng. Mater..

[5] Kolken H.M.A., Zadpoor A.A. (2017). Auxetic mechanical metamaterials. RSC. Adv..

[6] Ngo T.D., Kashani A., Imbalzano G., Nguyen K.T.Q., Hui D. (2018). Additive manufacturing (3D printing): A review of materials, methods, applications and challenges. Compos. B Eng..

[7] Gorhuluarslan R.M., Gandhi U.N., Mandapati R., Choi S.-K. (2015). Design and fabrication of periodic lattice-based cellular structure. Comput. Aided Des. Appl..

[8] Al-Ketan O., Rowshan R., Al-Rub R.K.A. (2018). Topology-mechanical property relationship of 3D printed strut, skeletal, and sheet based periodic metallic cellular materials. Addit. Manuf..

[9] McCaw J.C.S., Cuan-Urquizo E. (2018). Curved-layered additive manufacturing of non-planar, parametric lattice structures. Mater. Des..

[10] Kucewicz M., Baranowski P., Maƚachowski J., Popƚaswki A., Pƚatek P. (2018). Modelling, and characterization of 3D printed cellular structures. Mater. Des..

[11] Chen Y., Li Z., Jia Z., Scarpa F., Yao C.-W., Wang L. (2018). 3D printed hierarchical honeycombs with shape integrity under large compressive deformations. Mater. Des..

[12] Li T., Chen Y., Hu X., Li Y., Wang L. (2018). Exploiting negative Poisson’s ratio to design 3D-printed composites with enhanced mechanical properties. Mater. Des..

[13] Electrical Conductive Composite PLA. https://www.proto-pasta.com/collections/conductive/products/conductive-pla.

[14] Conductive Graphene PLA Filament. http://www.blackmagic3d.com/Conductive-p/grphn-pla.htm.

[15] Joshi A., Goh J.K., Goh E.J. (2020). 3D and 4D Printing of Polymer Nanocomposite Materials.

[16] Guo H., Lv R., Bai S. (2019). Recent advances on 3D printing graphene-based composites. Nano Mater. Sci..

[17] Espalin D., Muse D.W., MacDonald E., Wicker R.B. (2014). 3D printing multifunctionality: Structures with electronics. Int. J. Adv. Manuf. Technol..

[18] Kamyshny A., Magdassi S. (2019). Conductive nanomaterials for 2D and 3D printed flexible electronics. Chem. Soc. Rev..

[19] Athukorala S.S., Tran T.S., Balu R., Truong V.K., Chapman J., Dutta N.K., Choudhury N.R. (2021). 3D printable electrically conductive hydrogel scaffolds for biomedical applications: A review. Polymers.

[20] Ronca A., Rollo G., Cerruti P., Fei G., Gan X., Buonocore G.G., Lavorgna M., Xia H., Silvestre C., Ambrosio L. (2019). Selective laser sintering fabricated thermoplastic polyurethane/graphene cellular structures with tailorable properties and high strain sensitivity. Appl. Sci..

[21] Han Y., Lu W.F. (2018). Structural design of wearable electronics suitable for highly-stretched joint areas. Smart Mater. Struct..

[22] Wang Z., Gao W., Zhang Q., Zheng K., Xu J., Xu W., Shang E., Jiang J., Zhang J., Lu Y. (2019). 3D-printed graphene/polydimethylsiloxane composites for stretchable and strain-insensitive temperature sensors. ACS. Appl. Mater. Interfaces.

[23] Zhu C., Han Y.-J., Duoss E.B., Golobic A.M., Kuntz J.D., Spadaccini C.M., Worsley M.A. (2015). Highly compressible 3D periodic graphene aerogel microlattices. Nat. Commun..

[24] Kim H., Lee S. (2017). Effect on the electrical heating of textiles-coated graphene/waterborne polyurethane composites with different coating areas. Text. Sci. Eng..

[25] Filipowska B., Wiśniewski B., Michalak Z. (2018). Creation of electro-conductive paths and patterns by screen printing on textile bases. Text. Res. J..

[26] Kim H., Lee S. (2020). Characterization of electrical heating graphene/PLA honeycomb structure composite manufactured by CFDM 3D printer. Fash. Text..

[27] Kim H., Lee S. (2019). Characterization of electrical heating textile coated by graphene nanoplatelets/PVDF-HFP composite with various high graphene nanoplatelet contents. Polymers.

[28] Kim H., Lee S. (2019). Electrical heating properties of various electro-circuit patterns coated on cotton fabric using graphene/polymer composites. Text. Res. J..

[29] Kim H., Lee S. (2020). Characterization of electrical heating performance of CFDM 3D-printed graphene/polylactic acid (PLA) horseshoe pattern with different 3D printing directions. Polymers.

